# Bioassay-Guide Preparative Separation of Hypoglycemic Components from *Gynura divaricata* (L.) DC by Conventional and pH-Zone Refining Countercurrent Chromatography

**DOI:** 10.3390/foods14040578

**Published:** 2025-02-10

**Authors:** Zetao Shen, Jing Xu, Lijiao Wen, Lu Yin, Xueli Cao, Hairun Pei, Xi Zhao

**Affiliations:** 1 Beijing Advanced Innovation Center for Food Nutrition and Human Health, Beijing Technology and Business University, Beijing 100048, China; 2 Beijing Royal Integrative Medicine Hospital, Beijing 100027, China

**Keywords:** *Gynura divaricata* (L.) DC, high-speed countercurrent chromatography (HSCCC), pH-zone refining CCC, caffeoylquinic acids, hypoglycemic components

## Abstract

*Gynura divaricata* (L.) DC is a long-used medicinal and edible plant in China folk. Its hyperglycemic effects have garnered increasing public attention in recent years. This study revealed that the ethyl acetate (EtOAc) and butanol (BuOH) partition fractions of *G. divaricata* crude extract exhibited significantly higher α-glucosidase inhibition activity and enhanced glucose uptake ability compared to other fractions. Guided by the hypoglycemic bioassay, these two fractions were subjected to isolation of active compounds using high-speed countercurrent chromatography (HSCCC). A two-phase solvent system composed of hexane-methyl tert-butyl ether (MtBE)-methanol-0.1% TFA water was employed for the separation of the EtOAc fraction by conventional HSCCC through a gradient elution strategy. Five major compounds were obtained and identified as chlorogenic acid (1), 3,4-dicaffeoylquinic acid (2), 3,5-dicaffeoylquinic acid (3), 4,5-dicaffeoylquinic acid (4), and kaempferol-3-O-β-D-glucopyranoside (5) by ESI-MS, ^1^HNMR, and ^13^CNMR. The chlorogenic acid and the three dicaffeoylquinic acids were found to display higher inhibitory activities against α-glucosidase compared to the flavonoid. Considering their acidic nature, pH-zone-refining CCC (PHZCCC) was then applied for further scale-up separation using a solvent system MtBE: n-butanol: acetonitrile: water with trifluoroacetic acid (TFA) as a retainer and ammonium hydroxide (NH_4_OH) as an eluter. A significantly higher yield of chlorogenic acid was obtained from the BuOH fraction by PZRCCC. Molecular docking between the caffeoylquinic acids and α-glucosidase confirmed their hypoglycemic activities. This study demonstrates that CCC is a powerful tool for preparative separation of active constituents in natural products. This research presents a novel and effective method for the preparative isolation of hypoglycemic compounds from *Gynura divaricata.*

## 1. Introduction

*Gynura divaricata* (L.) DC (*G. divaricata*), commonly referred to as “Bai Bei San Qi” in China, is a valuable medicinal and edible plant distributed mainly in southern China. Both its stems and leaves have been long used in folk medicine for the treatment of diabetes and various other ailments [1]. In 2010, the China State Food and Drug Administration approved it as a new resource food.

In recent years, *Gynura divaricata* has been studied for various bioactivities, such as antioxidant, antihypertensive, and anti-cancer effects [2,3,4]. However, its anti-hyperglycemic effects have garnered increasing attention from researchers [5,6,7,8,9,10,11,12,13]. Most research focused on the hypoglycemic activity of *G. divaricata* extracts [5,6,7,8,9], while fewer studies have targeted its components, including polysaccharides, proteins, and phenolic acids. It has been reported that *G. divaricata* polysaccharides can regulate blood glucose potentially by directly inhibiting α-glucosidase, exerting antioxidant activity, and modulating intestinal microbiota [10]. A mixture of twenty-five diprotin A (a tripeptide) analogs isolated from *G. divaricata* was found exhibiting IC_50_ of 0.40 mg/mL for dipeptidyl peptidase IV (DPP-IV) [11]. The caffeoylquinic acid derivatives have been proposed as potentially responsible for the anti-diabetic activity of *G. divaricate* through the inhibition of yeast α-glucosidase and Protein Tyrosine Phosphatase 1B (PTP1B) in vitro [12]. *G. divaricata* powder rich in caffeoylquinic acids has been shown to successfully ameliorate hyperglycemia and hyperinsulinemia while improving the function of the pancreas [13]. However, the material basis and mechanism underlying its hypoglycemic activity remain unclear, and further intensive and systematic research is needed.

The reported methods for isolating and purifying components from *Gynura divaricata* typically involve the complicated column chromatographic separation, using silica gel, ODS, or Sephadex LH-20 [12,14]. High-speed countercurrent chromatography (HSCCC) is a liquid–liquid chromatographic separation technique in which both the stationary phase and the mobile phase are liquids, with no irreversible adsorption. It offers several advantages, including no loss of samples, no contamination, high efficiency, and rapid processing [15]. pH-zone-refining countercurrent chromatography (PZRCCC) is a specialized countercurrent chromatographic separation technology based on standard HSCCC. It achieves separation based on the acid dissociation constants (pKa) and hydrophobicity of compounds and is particularly applicable for the preparative separation of organic acids and bases. The preparative capacity of PZRCCC can be more than ten times greater than that of conventional CCC, and the target compounds obtained after separation are highly concentrated and pure [16]. While CCC has been applied to the isolation of phenolic acids from other plants, often combined with pre-enrichment using microporous resin adsorption or subsequent purification of preparative HPLC [17,18,19,20,21,22,23,24,25,26,27], CCC has not yet been used in the study of active phenolics in *G. divaricata*.

In this work, the hypoglycemic components of G. *divaricata* were isolated from its crude extract, guided by a bioassay of α-glucosidase inhibitory activity. Four active compounds were separated on a preparative scale using conventional HSCCC and PZRCCC. The structures of compounds were characterized by ESI-MS, ^13^C-NMR, and ^1^H-NMR, and their hypoglycemic activity was further confirmed by molecular docking.

## 2. Materials and Methods

### 2.1. Apparatus

CCC separation was performed on a HPCCC centrifuge (Dynamic Extraction, Uxbridge, UK). The apparatus has four columns on two bobbins all integrated into a single machine, with one analytical and one preparative column on each bobbin. The analytical columns are constructed with 0.8 mm internal diameter tubing forming a total column volume of 22.5 mL (11.0 and 11.5 mL). The preparative columns, using 1.6 mm internal diameter tubing, have a total column volume 133.5 mL (65.5 and 68.0 mL). The maximum rotational speed is 1600 rpm. A SH150-1500 constant temperature regulator (Lab Tech, Beijing, China) was employed to control the separation temperature. The HSCCC system was integrated with a Smartline HPLC system (KNAUER, Berlin, Germany), comprising a P-1000 pump, an UV-2500 detector, and a EuroChrom work station. The pH electrode S450C and the online pH test pool of FC49K were from Sensorex (Garden Grove, CA, USA).

The continuous wavelength microplate reader (SpectraMax190) was from Molecular Devices (San Jose, CA, USA).

The HPLC analyses were conducted using an Agilent 1100 HPLC system (Santa Clara, CA, USA), equipped with a quaternary pump, an auto-sampler, and a diode array detector (DAD). NMR analysis was performed on a Bruker AV 600 instrument (Rheinstetten, Germany), while electrospray mass spectrometry (ESI-MS) experiments were carried out on a Shimadzu LCMS-2010 (Kyoto, Japan) at the Institute of Chemistry, Chinese Academy of Sciences, Beijing.

### 2.2. Reagents and Materials

Analytical grade of n-butanol, acetonitrile, hexane, ethyl acetate, methanol, DMSO, ethanol, petroleum, methyl tert-butyl ether (MtBE), ammonium hydroxide (NH_4_OH), and trifluoroacetic acid (TFA) were purchased from Beijing chemical factory (Beijing, China). Glucose assay kit (GOD-POD method) was purchased from BioSino Bio-Technology & Science Inc. (Beijing, China). A 0.9% saline solution was purchased from China Resources Shuanghe Pharmaceutical Co. (Beijing, China). Phosphate buffer PBS, α-glucosidase (Rat intestinal acetone powder), and insulin were all purchased from Shanghai Macklin Biochemical Technology Co. (Shanghai, China). Methanol and acetic acid used for HPLC analysis was of chromatographic grade and purchased from Fisher Chemical (Loughborough, UK). Water was produced through a Milli-Q system (18 MΩ) (Millipore, MA, USA).

The dried leaves of *G. divaricata* were obtained from Shanxi Pujing Biotechnology Co., Ltd., Taiyuan, China.

### 2.3. Extraction of Gynura divaricata

The leaves of *G. divaricata* (1.5 kg, dry weight) were extracted with 10 L of 65% ethanol four times, each for 48 h, under maceration in a 20 L glass bottle at room temperature. After combining the extracts and removing the solvent under vacuum, 400 mL ethanol-free extract solution was obtained. It was then extracted successively with petroleum ether, ethyl acetate (EtOAc), and *n*-butanol (BuOH). After concentration and drying, 39.57 g of petroleum ether fraction, 12.96 g of EtOAc fraction, 27.54 g of BuOH fraction, and 84.37 g of water fraction were obtained and temporarily stored in a refrigerator at 4 °C. The fractions were tested for their inhibitory activity on α-glucosidase and their effect on glucose uptake on human hepatocellular carcinoma (HepG2) cells. Guided by the bioassay, the EtOAc and BuOH extract were subjected to further separation and purification of hypoglycemic components through conventional CCC and PZRCCC.

### 2.4. Bioassay of Extracts

#### 2.4.1. α-Glucosidase Inhibition Assay

The α-glucosidase solution was prepared by dissolving 1 g of α-glucosidase powder in 20 mL of 0.9% saline solution under ultrasonication in an ice bath, and the supernatant was collected after centrifugation. The sample solution was prepared by dissolving in DMSO at a different concentration. The assay included five groups: blank control, negative control, positive control, sample, and sample control, each with three replicates (Table S1). In each well of 96-well plate, added α-glucosidase, PBS buffer (pH 6.8), and sample solution sequentially. The plate was incubated at 37 °C for 15 min before adding the substrate solution (0.5 mol/L sucrose solution), followed by an additional 30 min incubation. After incubation, 150 μL of 0.1 mol/L Na_2_CO_3_ was added to each well to stop the reaction. Then, 10 μL of each aborted reaction solution was transferred to another enzyme plate, 200 μL of glucose assay kit solvent was added, and the plate was incubated at 37 °C for 15 min. Absorbance at 505 nm was measured using a continuous-wavelength microplate reader. The inhibition rate was calculated using Formula (1):

(1) $$\alpha -Glucosidase\;Inhibition\;rate=\frac{\left({OD}_{negative\;control}-{OD}_{blank\;control}\right)-\left({OD}_{sample}-{OD}_{sample\;control}\right)}{{OD}_{negative\;control}-{OD}_{blank\;control}}\times 100\%$$

#### 2.4.2. Glucose Uptake Assay

HepG2 cells were washed twice with the test solution before being seeded into a 96-well plate and cultured for about 24 h at 37 °C in a 5% CO_2_ incubator. The test samples or insulin (final concentration 30 nM, positive control) were then added and incubated at 37 °C for 2 h. The glucose concentration in the supernatant was measured using a glucose oxidase–peroxidase coupling enzyme (GOD-POD) method at 505 nm with a microplate reader. The glucose uptake rate was calculated using Formula (2):

(2) $$Glucose\;uptake\;rate=\frac{{OD}_{blank\;control}-{OD}_{sample}}{{OD}_{blank\;control}-{OD}_{background}}\times 100\%$$

### 2.5. CCC Separation Procedure

For conventional HSCCC, the column was first filled with the upper stationary phase; then, the lower mobile phase was pumped into the column from head to tail at a flow rate of 1.0 or 1.5 mL/min (for 22.5 mL analytical column) or 6.0 mL/min (for 133.5 mL preparative column), while the rotor rotated at 1600 rpm. Once equilibrium established, the sample solution was introduced into the column via the sample valve. The effluent was continuously monitored with a UV detector at 254 nm. Peak fractions were manually collected according to the chromatogram. Each fraction was evaporated under reduced pressure and re-dissolved in methanol for the HPLC analysis.

For PZRCCC, the column was entirely filled with the organic stationary phase containing a retainer acid, which was followed by a sample injection. Then, the aqueous mobile phase containing an eluter base was pumped into the column at a flow rate of 2.0 mL/min while the column rotated at 1600 rpm. The effluent from the column was continuously monitored at 254 nm and collected into test tubes at 3 min intervals (6.0 mL/tube) using a fraction collector. The pH was monitored on-line with a pH detector connected with a flow cell.

### 2.6. HPLC Analysis and Elucidation of Isolated Compounds

The extracts and fractions separated by CCC were analyzed using an Agilent 1100 HPLC system equipped with an Ultimate XB-C18 column (250 mm × 4.6 mm i.d., 5 μm, Welch Materials, Concord, MA, USA). The mobile phase consisted of methanol (A) and 2% acetic acid aqueous solution (B) in the gradient elution mode: 0–10 min, 25% A; 10–15 min, 25–40% A; 15–20 min, 40% A; 20–30 min, 40–60% A; 30–35 min, 60–100% A; 35–45 min, 100% A. The flow rate was 1.0 mL/min, and the temperature was set to 30 °C. The detection wavelength was 254 nm, and the injection volume was 10 μL.

The chemical structures of isolated compounds were determined using electrospray ionization mass spectrometry (ESI-MS) and NMR techniques. Mass spectrometry was conducted under the following conditions: the ESI source had a mass-to-charge ratio range of 50 to 2200 m/z. The atomizing gas pressure was 40 psi, the drying gas flow rate was 10 L/min, the drying gas temperature was 350 °C, and the sheath gas (N_2_) flow rate was 1.5 L/min. The mobile phase consisted of methanol and water (1:1, *v*/*v*), with a flow rate of 0.07 mL/min. The ionization mode was negative ion mode. ^1^H-NMR and ^13^C-NMR analysis were performed under 600 MHz and 150 MHz, respectively, in DMSO-*d_6_* with tetramethylsilane (TMS) as the reference. The temperature was set at 25 °C.

### 2.7. Molecular Docking

The isolated compounds were evaluated for their interaction with α-glucosidase through molecular docking. The three-dimensional structures of the five compounds were geometrically optimized and energy minimized using AutoDock tools v1.5.6 and OpenBabel-2.3.2 software. The crystal structure of α-glucosidase (PDB ID: 3W37) [28] was obtained from the Protein Data Bank (PDB, https://www.rcsb.org/). The protein structure underwent several processing steps, including the removal of the original ligand and water molecules, the addition of polar hydrogen atoms, and charge calculation and assignment using PyMOL v2.6.0a0. Docking analyses were finally conducted using AutoDock Vina v1.1.2 with default parameter, and the molecular docking results were visualized using PyMOL v2.6.0a0.

## 3. Results and Discussion

### 3.1. The Hypoglycemic Effect of Different Solvent Fractions

**Figure 1:**
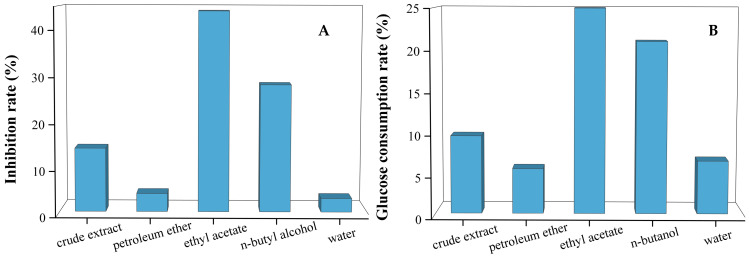
The hypoglycemic activity of different fractions of the crude extract of *G. divaricata*. α-glucosidase inhibition activities (**A**); effect on HepG2 glucose uptake (**B**).

The four solvent fractions of the crude extract of G. divaricata were evaluated for their hypoglycemic effects. As shown in Figure 1, the EtoAc and BuOH fractions exhibited significantly higher α-glucosidase inhibition activity (A) compared to petroleum ether and residual water fractions at the same concentration. The positive glucose uptake rates (B) of the crude extract and its four sub-fractions indicated their varying promotion effect on glucose uptake, with the ethyl acetate and n-butanol fractions significantly higher than the others. Based on these findings, the EtOAc and BuOH fractions were selected for further investigation of their active components.

### 3.2. HPLC Analysis of EtOAc and BuOH Fractions

**Figure 2:**
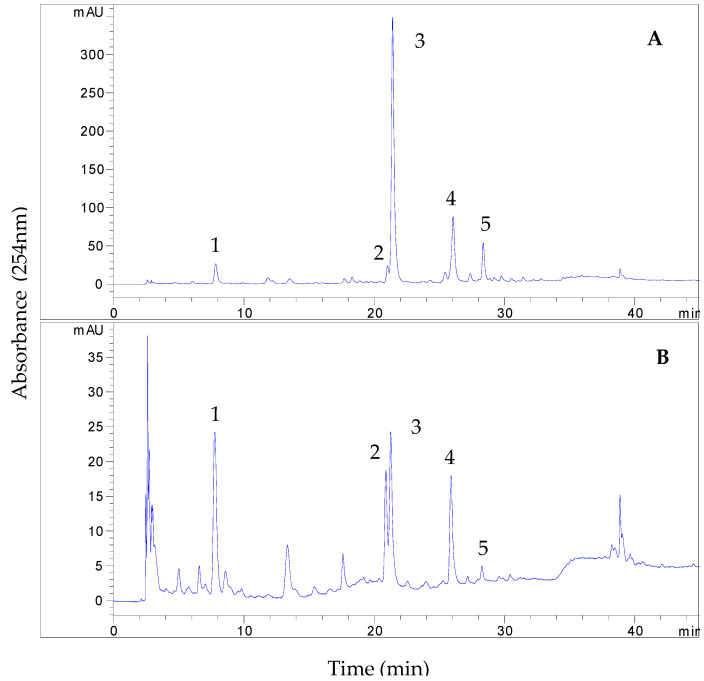
HPLC chromatograms of the EtOAc (**A**) and BuOH (**B**) fractions of the *G. divaricata* extract.

Figure 2 presents the HPLC profiles of the two fractions of the *G. divaricata* extract. Both chromatograms displayed five prominent peaks. Peaks 1–4 exhibited similar UV spectra, with absorbance maxima around 245 nm, 300 nm, and 325 nm, suggesting they may be phenolic acids. Peak 5 displayed a distinct spectrum with two absorbance maxima at 250–280 nm and 304–360 nm, characteristic of flavonoids. Based on these observations, the five peaks were traced as the potential target compounds in the following separation.

### 3.3. Conventional CCC Separation of the EtOAc Fraction

Due to differences in composition and interferences, various solvent systems must be employed for the separation of samples from different matrixes. Based on the literature reports, the classic HEMWat system, consisting of hexane–ethyl acetate–methanol–water, was evaluated in a series of volume ratios ranging from 1:1:1:1 to 1:9:1:9, as well as different elution modes for the initial isolation of potential active compounds in the ethyl acetate fraction using the analytical column of CCC. However, satisfactory resolution of all targets within an acceptable separation time was not achieved.

**Figure 3:**
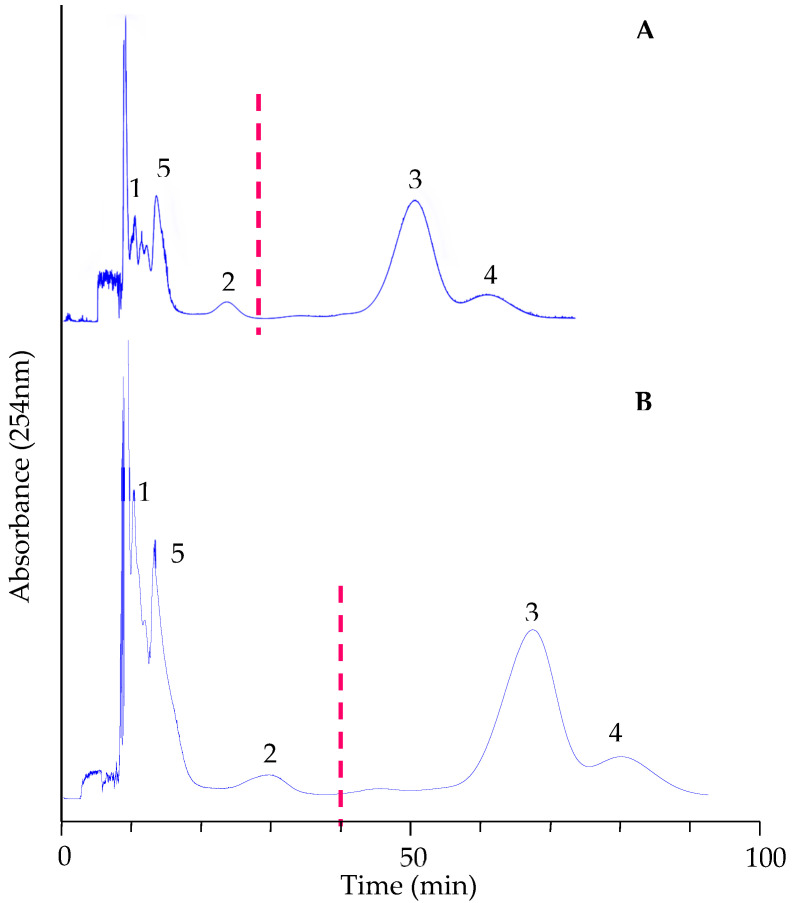
Analytical (**A**) and preparative (**B**) CCC chromatograms of the ethyl acetate fraction of *G. divaricata* extract under the optimized conditions. Mobile phase: gradient from the lower phase of hexane–MtBE–methanol–0.1% TFA water (0:10:1:9) to (2:8:2:8); flow rate: 1.5 mL/min (**A**) and 6.0 mL/min (**B**); revolution speed: 1600 rpm; column temperature: 30 °C; detection: 254 nm; sample: 20 mg/1 mL (**A**) and 120 mg/6 mL (**B**). The peak 1–5 are corresponding to the peak 1–5 in Figure 2.

To improve separation, the HEMWat system was modified by replacing ethyl acetate with methyl tert-butyl ether (MtBE) and adding 0.1% TFA to the water, forming a new solvent system: hexane–MtBE–methanol–0.1% TFA water (1:9:1:9, *v*/*v*) (modified 1). Further optimization led to the selection of a gradient elution strategy, transitioning from hexane–MtBE–methanol–0.1% TFA water (0:10:1:9) (modified 2) to 2:8:2:8 (modified 3), which was applied in preparative separation. Figure 3 shows the separation profiles for the analytical (A) and preparative (B) scales.

In the preparative separation, the sample loading was scaled up in proportion to the column volume (133.5 mL vs. 22.5 mL). The sample loading was increased to 6.0 mL (20 mg/mL sample solution). To maintain the same separation time, the flow rate of mobile phase should also scale up six times, from 1.5 mL/min to 9.0 mL/min. However, considering the column pressure, a flow rate of 6.0 mL/min was employed. Consequently, the preparative separation time (100 min) was slightly longer than the analytical scale (80 min).

**Figure 4:**
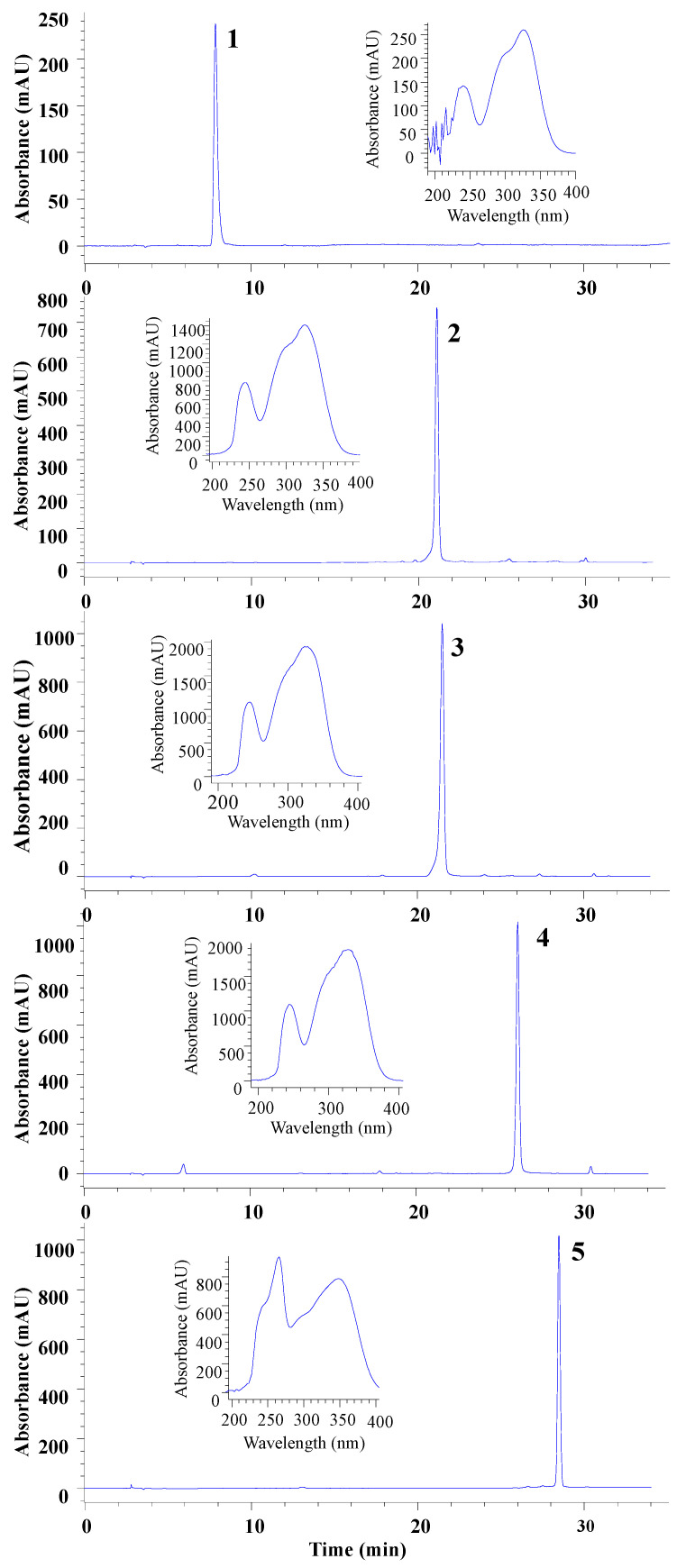
HPLC analysis of the compounds isolated from preparative CCC separation of ethyl acetate fraction.

As shown in Figure 3, compounds in peaks 1 and 5 were still not well resolved from the others, so they were subjected to further purification using the solvent system ethyl acetate–0.1% TFA water (1:1). Finally, five pure compounds were obtained from a 250 mg ethyl acetate extract of *G. divaricata*: 6.5 mg of compound **1** (98.54% HPLC purity), 3.5 mg of compound **2** (96.62% purity), 38.6 mg of compound **3** (97.14% purity), 7.3 mg of compound **4** (96.55% purity), and 24.4 mg of compound **5** (98.51% purity). Their individual UV spectra were identical to those in the initial sample. Figure 4 shows the purity analysis of these compounds.

**Figure 5:**
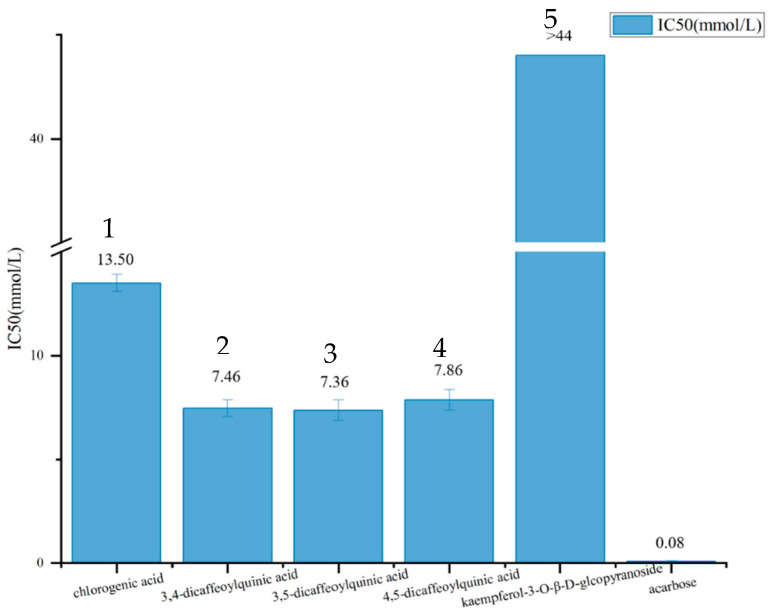
α-glucosidase inhibitory activities of isolated compounds.

The five isolated compounds were dissolved in DMSO at different concentration, and their IC_50_ values for α-glucosidase inhibition were measured, as shown in Figure 5. Compounds **2**–**4** exhibit significantly higher inhibitory activities, followed by compound **1**, while compound **5** shows the lowest activity.

### 3.4. Structure Identification of Pure Compounds

**Figure 6:**
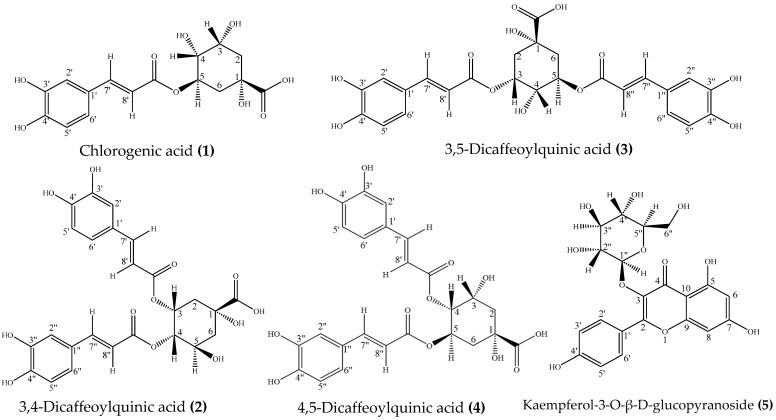
The structures of the five identified compounds.

The structure identification of five compounds (**1**–**5**) was performed with ESI-MS, ^1^H NMR (600 MHz), and ^13^C NMR (150 MHz). Five compounds (**1**–**5**) were tentatively identified as chlorogenic acid (**1**), 3,4-dicaffeoylquinic acid (**2**), 3,5-dicaffeoylquinic acid (**3**), 4,5-dicaffeoylquinic acid (**4**), and kaempferol-3-O-β-D-glucopyranoside (**5**) by δ and J values analysis, and comparison with references [18,22,29]. ESI-MS measurements supported this identification, exhibiting [M-H]^-^ ions at m/z 353.2 (**1**, C_16_H_18_O_9_), m/z 515.2 (**2**, **3**, **4**, C_25_H_24_O_12_), and m/z 447.2 (**5**, C_21_H_20_O_11_). The chemical structures of the five identified compounds were illustrated in Figure 6. The NMR data for each compound are provided below, with the spectra available in Figure S5. The assignments, especially for the three dicaffeoylquinic acid isomers, can be further confirmed using 2D-NMR analysis.

Chlorogenic acid (**1**): ^1^H NMR (DMSO-*d*_6_): δ_H_ 7.43 (1H, d, 15.6 Hz, H-7’), 7.04 (1H, d, 1.8 Hz, H-2’), 6.99 (1H, dd, 7.8, 1.8 Hz, H-6’), 6.78 (1H, d, 7.8 Hz, H-5’), 6.15 (1H, d, 15.6 Hz, H-8’), 5.07 (1H, m, H-5), 3.94 (1H, m, H-3), 3.57 (1H, m, H-4), 2.00–2.04 (2H, m, H-6), 1.77–1.96 (2H, m, H-2). ^13^C NMR (DMSO-d6): δ_C_ 175.37 (-COOH), 165.17 (-COO), 148.78 (C-4’), 146.01 (C-3’), 145.39 (C-7’), 126.04 (C-1’), 121.80 (C-6’), 116.19 (C-2’), 115.22 (C-5’), 114.73 (C-8’), 73.90 (C-1), 71.32 (C-5), 70.79 (C-3), 68.48 (C-4), 37.65 (C-6), 36.66 (C-2).

3,4-Dicaffeoylquinic acid (**2**): ^1^H NMR (DMSO-*d*_6_): δ_H_ 7.43 (1H, d, 15.6 Hz, H-7’), 7.43 (1H, d, 15.6 Hz, H-7”), 6.99 (1H, d, 1.8 Hz, H-2’), 6.99 (1H, d, 1.8 Hz, H-2”), 6.98 (1H, dd, 7.8 Hz, 1.8 Hz, H-6’), 6.97 (1H, dd, 7.8 Hz, 1.8 Hz, H-6”), 6.71 (1H, d, 7.8 Hz, H-5’), 6.68 (1H, d, 7.8 Hz, H-5”), 6.19 (1H, d, 15.6 Hz, H-8’), 6.15 (1H, d, 15.6 Hz, H-8”), 5.37 (1H, m, H-3), 4.88 (1H, m, H-4), 4.06 (1H, m, H-5), 2.14 (2H, m, H-6), 1.90 (2H, m, H-2). ^13^C NMR (DMSO-*d*_6_): δ_C_ 176.14 (-COOH), 166.43 (-COO), 166.28 (-COO), 148.89 (C-4”), 148.80 (C-4’), 146.04 (C-7”), 146.04 (C-7’), 145.75 (C-3”), 145.57 (C-3’), 125.98 (C-1”), 125.90 (C-1’), 121.87 (C-6”), 121.77 (C-6’), 116.22 (C-5”), 116.22 (C-5’), 115.26 (C-2”), 115.13 (C-2’), 114.73 (C-8”), 114.42 (C-8’), 73.18 (C-1), 68.83 (C-3), 68.83 (C-4), 64.68 (C-5), 37.58 (C-6), 36.02 (C-2).

3,5-Dicaffeoylquinic acid (**3**): ^1^H NMR (DMSO-*d*_6_): δ_H_ 7.49 (1H, d, 15.6 Hz, H-7’), 7.45 (1H, d, 15.6 Hz, H-7”), 7.05 (1H, d, 1.8 Hz, H-2’), 7.04 (1H, d, 1.8 Hz, H-2”), 6.99 (1H, dd, 7.8 Hz,1.8 Hz, H-6’), 6.99 (1H, dd, 7.8 Hz, 1.8 Hz, H-6”), 6.79 (1H, d, 7.8 Hz, H-5’), 6.78 (1H, d, 7.8 Hz, H-5”), 6.26 (1H, d, 15.6 Hz, H-8’), 6.16 (1H, d, 15.6 Hz, H-8”), 5.20 (1H, m, H-5), 5.12 (1H, m, H-3), 3.85 (1H, brs, H-4), 2.15 (2H, m, H-6), 1.98 (2H, m, H-2). ^13^C NMR (DMSO-*d*_6_): δ_C_ 175.67 (-COOH), 166.53 (-COO), 165.99 (-COO), 148.85 (C-4”), 148.73 (C-4’), 146.04 (C-7”), 146.04 (C-7’), 145.61 (C-3”), 145.23 (C-3’), 126.10 (C-1”), 126.03 (C-1’), 121.86 (C-6”), 121.62 (C-6’), 116.27 (C-5”), 116.21 (C-5’), 115.29 (C-2”), 115.21 (C-2’), 114.56 (C-8”), 114.56 (C-8’), 72.88 (C-1), 71.33 (C-3), 70.93 (C-5), 67.78 (C-4), 35.96 (C-6), 35.06 (C-2).

4,5-Dicaffeoylquinic acid (**4**): ^1^H NMR (DMSO-*d*_6_): δ_H_ 7.49 (1H, d, 15.6 Hz, H-7’), 7.43 (1H, d, 15.6 Hz, H-7”), 7.03 (1H, d, 1.8 Hz, H-2’), 7.02 (1H, d, 1.8 Hz, H-2”), 6.99 (1H, dd, 7.8 Hz, 1.8 Hz, H-6’), 6.99 (1H, dd, 7.8 Hz, 1.8 Hz, H-6”), 6.76 (1H, d, 7.8 Hz, H-5’), 6.74 (1H, d, 7.8 Hz, H-5”), 6.25 (1H, d, 15.6 Hz, H-8’), 6.15 (1H, d, 15.6 Hz, H-8”), 5.35 (1H, m, H-5), 4.97 (1H, dd, 7.6, 2.7, H-4), 4.18 (1H, m, H-3), 2.14–2.18 (4H, m, H-6, H-2). ^13^C NMR (DMSO-*d*_6_): δ_C_ 175.08 (-COOH), 166.42 (-COO), 165.97 (-COO), 148.93 (C-4”), 148.93 (C-4’), 146.01 (C-7”), 146.01 (C-7’), 145.95 (C-3”), 145.95 (C-3’), 125.89 (C-1”), 125.89 (C-1’), 121.92 (C-6”), 121.80 (C-6’), 116.25 (C-5”), 116.18 (C-5’), 115.59 (C-2”), 115.32 (C-2’), 114.32 (C-8”), 114.05 (C-8’), 73.90 (C-1), 68.08 (C-4), 67.30 (C-5), 66.63 (C-3), 37.94 (C-6), 36.12 (C-2).

Kaempferol-3-O-β-D-glucopyranoside (**5**): ^1^H NMR (DMSO-*d*_6_): δ_H_ 8.00 (2H, d, 9.0 Hz, H-2’, 6’), 6.84 (2H, d, 9.0 Hz, H-3’, 5’), 6.39 (1H, d, 2.4 Hz, H-8), 6.17 (1H, d, 2.4 Hz, H-6), 5.42 (1H, d, 7.8 Hz, H-1”), 3.47–3.53 (2H, m, H-6”), 3.13–3.18 (2H, m, H-2”, 3”), 3.04 (2H, m, H-4”, 5”). ^13^C NMR (DMSO-*d*_6_): δ_C_ 177.94 (C-4), 164.59 (C-7), 161.70 (C-5), 160.42 (C-4’), 156.85 (C-9), 156.72 (C-2), 133.66 (C-3), 131.34 (C-2’, 6’, d), 121.36 (C-1’), 115.56 (C-3’,5’, d), 104.48 (C-10), 101.34 (C-1”), 99.14 (C-6), 94.10 (C-8), 79.43 (C-2”), 77.96 (C-5”), 74.68 (C-3”), 70.36 (C-4”), 61.31 (C-6”).

### 3.5. PZRCCC Separation of Four Phenolic Acids in Large-Scale

Considering the acidic nature of phenolic acid compounds, which are the primary components in both the ethyl acetate and butanol fractions, PHZCCC can be utilized for preparative separation of these compounds in a large scale. To effectively separate acidic compounds, a solvent system with optimal partition coefficients (K) in both acidic (Kacid >> 1) and basic (Kbase << 1) conditions, along with sufficient sample solubility, is required. A two-phase solvent system containing *n*-butanol was tested to achieve better resolution of polar phenolic acids. The solvent system composed of MtBE: *n*-butanol: acetonitrile: water in the volume ratio of 2:2:1:5 and 1:3:1:5 (*v*/*v*), with TFA (10 mM, pH = 2) as the retainer in the upper stationary phase and NH_4_OH (10 mM, pH = 10.0) as the eluter in the lower mobile phase, was evaluated for the K values of the target phenolic acids. As shown in Table 1, the system with a ratio of 1:3:1:5 demonstrated a more suitable Kacid value of compound 1, in accordance with “the golden rule” suggested by Ito [16].Therefore, it was employed for the subsequent PZRCCC separation of phenolic acids from the ethyl acetate and butanol extracts.

**Figure 7:**
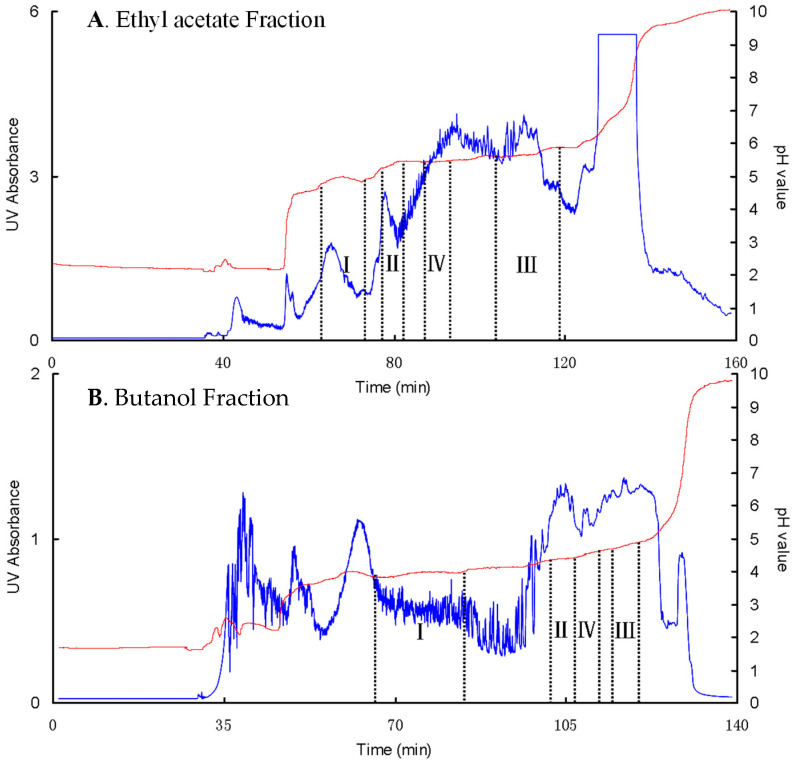
PZRCCC of the ethyl acetate and butanol fractions of G. divaricate extract. Solvent system: MtBE: n-butanol: acetonitrile: water (1:3:1:5, *v*/*v*), TFA (10 mM, pH = 2) as the retainer in the upper stationary phase and NH_4_ OH (10 mM, pH = 10.0) as the eluter in the lower mobile phase; flow rate: 2.0 mL/min; revolution speed: 1600 rpm; column temperature: 30 °C; detection: 254 nm; sample amount: 1 g.

In the PZRCCC separation, the four rectangular zones (I–IV) marked in Figure 7 correspond to the chromatographic peaks of four phenolic acids detected by HPLC as chlorogenic acid (I, **1**), 3,4-dicaffeoylquinic acid (II, **2**), 3,5-dicaffeoylquinic acid (III, **3**), and 4,5-dicaffeoylquinic acid (IV, **4**). However, the elution order of compound 3 and 4 was reversed to the HPLC results, which is consistent with previous reports in the literature [22]. With the increase in loading sample amount from 500 mg to 1 g, the width of each zone broadened, and the elution time increased accordingly without significant loss of compound purity (Figures S1 and S2, Table S2). The isolated compounds mainly concentrate in each zone in high purity, while the interferences are accumulated at the zone boundaries, highlighting the advantage of PZRCC over conventional CCC. After combining the high-purity fractions, concentrating and freeze-drying, 25.9 mg of chlorogenic acid (95.21% purity), 29.4 mg of 3,4-dicaffeoylquinic acid (91.00% purity), 89.2 mg of 3,5-dicaffeoylquinic acid (94.48% purity), and 36.5 mg of 4,5-dicaffeoylquinic acid (90.70%) were obtained from 1.0 g of the ethyl acetate extract.

The elution order and the resolution of the compound zones in PZRCC are determined by both the ${pK}_{a}$ and hydrophobicity. According to Ito’s theory [16], two ionic solutes can be resolved if the difference in their zone pH (${pH}_{z-s}$) exceeds 0.2. The ${pH}_{z-s}$ is calculated as Equation (3), where $K_{D-S}$ represents the partition ratio of solute S (RCOOH) in zone S (calculated as Equation (4)), which reflects its hydrophobicity. $K_{s}$ is the partition coefficient of the solute (calculated as Equation (5)). ${pK}_{a}$ is a constant specific to the solute, $K_{D-S}$ is a constant when the solvent system is selected, and $K_{s}$ is a variable that depends on the pH of the solvent system. Therefore, two strategies can be employed to increase the ${pH}_{z-s}$ difference between adjacent solute zones in PZRCC: one is to modify the composition of two-phase solvent systems, and the other is to adjust the concentration of the retainer acid and eluter base.

(3) $${pH}_{z-s}={pK}_{a}+log\{\left(\frac{K_{D-S}}{K_{s}}\right)-1\}$$

(4) $$K_{D-S}=\frac{\left[{RCOOH}_{ORG}\right]}{{[RCOOH}_{AQ}]}$$

(5) $$K_{S}=\frac{\left[{RCOOH}_{ORG}\right]}{{[RCOOH}_{AQ}]+{[RCOO}_{AQ}^{-}]}$$

In this study, the ${pK}_{a}$ of chlorogenic acid (I, 3.11) [30] is quite different from that of 3,4-dicaffeoylquinic acid (II, 3.69), 3,5-dicaffeoylquinic acid (III, 3.52), and 4,5-dicaffeoylquinic acid (IV, 3.69) (values obtained from SciFinder). As a result, chlorogenic acid was eluted earlier with a relatively higher purity, while the three dicaffeoylquinic acid isomers with similar pKa values exhibited a slight overlap with each over. Their resolution could potentially be improved through further optimization of the solvent system composition and the concentrations of acid and base.

The separation of *n*-butanol fraction by PZRCCC resulted in a higher yield of chlorogenic acid as shown in Figure 7. However, due to the more complicated composition of butanol fraction, as illustrated in Figure 7B, three dicaffeoylquinic acid isomers were achieved in limited amounts with high purity (seen also in Table S2). A 1.0 g sample of the *n*-butanol fraction yielded 65 mg of chlorogenic acid (92.54% purity), 24.5 mg of 3,4-dicaffeoylquinic acid (95.67% purity), 9.3 mg of 3,5-dicaffeoylquinic acid (86.63% purity), and 4.7 mg of 4,5-dicaffeoylquinic acid (87.70%). The second purification step was tentatively employed, resulting in higher purity (>98%) of dicaffeoylquinic acid isomers (seen in Figure S3).

### 3.6. Molecular Interaction Between the Isolated Compounds and α-Glucosidase

**Figure 8:**
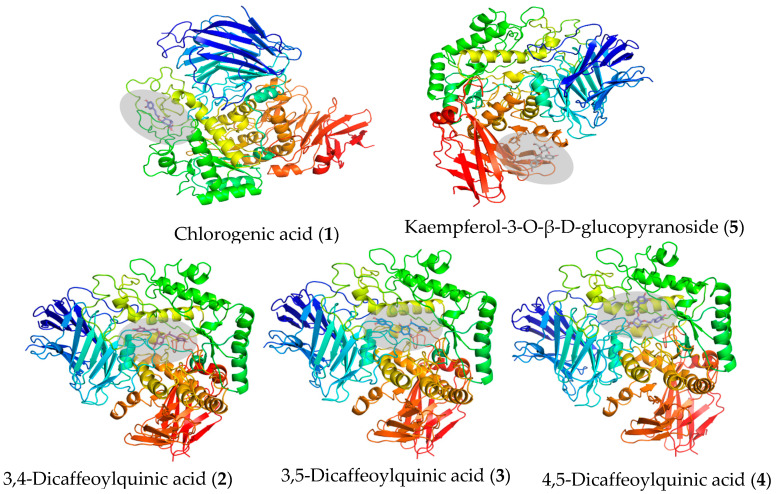
Molecular interaction between the isolated compounds and α-glucosidase.

The five isolated compounds were evaluated for their inhibitory effects on α-glucosidase through molecular docking. Higher docking scores (binding energy values) indicate more stable and rational interactions between the ligand and the protein. As illustrated in Figure 8, the three dicaffeoylquinic acids (**2, 3, 4**) were stably docked into the active site of α-glucosidase, in the same pocket where acarbose binds, as reported in the literature [28]. They combined with the target protein through amino acid residues similar to those of acarbose, which is notably different from the interaction of chlorogenic acid (**1**) and kaempferol-3-O-β-D-glucopyranoside (**5**), as seen in Table 2 and Figure S4. The differences in the interaction bonds between the specific molecules and the distinct amino acid residues of α-glucosidase resulted in varying inhibitory activities. This supports the α-glucosidase inhabitation assay results mentioned earlier.

## 4. Conclusions

In this work, the ethyl acetate and butanol partition fractions of *G. divaricata* extract, which exhibited higher hypoglycemic activity, were subjected to the isolation of active compounds using HSCCC. The separation of the ethyl acetate fraction by conventional HSCCC, employing a gradient elution strategy with a system hexane–MtBE–methanol–0.1% TFA water from 0:10:1:9 to 2:8:2:8 (*v*/*v*) produced five major compounds, including chlorogenic acid (HPLC purity, 98.54%), 3,4-dicaffeoylquinic acid (96.62%), 3,5-dicaffeoylquinic acid (97.14%), 4,5-dicaffeoylquinic acid (96.55%), and kaempferol-3-O-β-D-glucopyranoside (98.51%). They were identified using ESI-MS, ^1^HNMR, and ^13^CNMR. The caffeoylquinic acids, particularly the three dicaffeoylquinic acid isomers, were found to display remarkably higher inhibitory activities against α-glucosidase compared to the flavonoid. Given their acidic nature, PHZCCC was employed for further scale-up separation using a solvent system composed of MtBE: n-butanol: acetonitrile: water (1:3:1:5, *v*/*v*) with TFA (10 mM, pH = 2) as the retainer in the upper stationary phase and NH_4_OH (10 mM, pH = 10.0) as the eluter in the lower mobile phase. From 1.0 g of the ethyl acetate extract, 25.9 mg of chlorogenic acid (95.21%), 29.4 mg of 3,4-dicaffeoylquinic acid (91.00%), 89.2 mg of 3,5-dicaffeoylquinic acid (94.48%), and 36.5 mg of 4,5-dicaffeoylquinic acid (90.70%) were obtained in a single run, while the separation of the *n*-butanol fraction by PZRCCC yielded significantly more chlorogenic acid. Molecular docking studies between the caffeoylquinic acids and α-glucosidase confirmed their hypoglycemic activities. This study provided a new and efficient approach for the preparative separation of hypoglycemic compounds from *Gynura divaricate*, it has the potential to be scaled up in industrial processes, where the solvent used must meet food safety standards.

## Figures and Tables

**Table 1:** Partition coefficients of target phenolic acids in different solvent systems of pH-zone-refining CCC.

Solvent Systems (*v*/*v*)	Compound (1)		Compound (2)		Compound (3)		Compound (4)	
	K_acid_	K_base_	K_acid_	K_base_	K_acid_	K_base_	K_acid_	K_base_
MtBE: n-butanol: acetonitrile: water (2:2:1:5, *v*/*v*)	1.37	0.01	3.15	0.03	3.27	0.03	6.64	0.03
MtBE: n-butanol: acetonitrile: water (1:3:1:5, *v*/*v*)	4.71	0.01	4.60	0.05	15.14	0.08	20.74	0.03

**Table 2:** Molecular docking information of the isolated compounds with the α-glucosidase.

Compounds	Hydrogen Bonds	Binding Affinity (kcal/mol)
Chlorogenic acid	ASP 423, GLU 424, ASN 417, ASP 440	−7.6
Kaempferol-3-O-β-D-glucopyranoside	ASP 684, THR 681, ARG 699	−8.5
3,4-Dicaffeoylquinic acid	HIS 626, ALA 234, SER 497	−9.7
3,5-Dicaffeoylquinic acid	HIS 626, ALA 234, SER 497 ASP 357, ASP 232, ASN 496, SER 505	−9.2
4,5-Dicaffeoylquinic acid	ALA 234, ASP 357	−8.9
Acarbose *	HIS 626, ASP 357, ASP 568, ARG 522, ASP 232, ASN 237, SER 497	

* The data for acarbose are from reference [28].

## Data Availability

The original contributions presented in this study are included in the article/Supplementary Materials. Further inquiries can be directed to the corresponding author.

## Supplementary Materials

The following supporting information can be downloaded at: https://www.mdpi.com/article/10.3390/foods14040578/s1, Table S1: Experiment array (Unit: μL); Table S2: Yield and purity of separated compounds by pH-Zone-refining CCC. Figure S1: pH-zone-refining CCC of the ethyl acetate fraction of G. divaricate extract. Solvent system: MtBE: n-butanol: acetonitrile: water (1:3:1:5, *v*/*v*), TFA (10 mM, pH = 2) as retainer in the upper stationary phase and NH_4_OH (10 mM, pH = 10.0) as eluter in the lower mobile phase; Flow-rate: 2.0 mL/min; Revolution speed: 1600 rpm; Column temperature: 30 °C; Detection: 254 nm. Figure S2: pH-zone-refining CCC of the butanol fraction of G. divaricate extract. The conditions were the same as in Figure S1. Figure S3: Second purification of three dicaffeoylquinic acids by pH-zone-refining CCC. The conditions are the same with Figures S1 and S2. Figure S4: Molecular docking sites between the isolated compounds and the α-glucosidase. Figure S5-1: 1HNMR and 13CNMR spectrum of Compound **1**. Figure S5-2: 1HNMR and 13CNMR spectrum of Compound **2**. Figure S5-3: 1HNMR and 13CNMR spectrum of Compound **3**. Figure S5-4: 1HNMR and 13CNMR spectrum of Compound **4**. Figure S5-5: 1HNMR and 13CNMR spectrum of Compound **5**.
