# Supplementary material for: Bioassay-Guide Preparative Separation of Hypoglycemic Components from Gynura divaricata (L.) DC by Conventional and pH-Zone Refining Countercurrent Chromatography
Source: Foods. 2025 Feb 10;14(4):578. doi: 10.3390/foods14040578 (PMC11854274; doi:10.3390/foods14040578)
Supplement: Supplementary file 1 [file foods-14-00578-s001.zip › foods-3371758-supplementary.pdf]

Supplementary

Bioassay-Guide Preparative Separation of Hypoglycemic Components from *Gynura divaricata* (L.) DC by Conventional and pH-Zone Refining Countercurrent Chromatography

Zetao Shen [1], Jing Xu [1,2], Lijiao Wen [1], Lu Yin [1], Xueli Cao [1,*], Hairun Pei [1] and Xi Zhao [1]

[1] Beijing Advanced Innovation Center for Food Nutrition and Human Health, Beijing Technology and Business University, Beijing 100048, China
[2] Beijing Royal Integrative Medicine Hospital, Beijing 100027, China
* Correspondence: caoxl@th.btbu.edu.cn

Table S1. Experiment array (Unit: μL)

Groups	α-Glucosidase	PBS buffer	DMSO	Sample	Substrate
Blank control	25(Inactivate)	75	25		25
Negative control	25	75	25		25
Positive control (Acarbose)	25	75		25	25
Sample be tested	25	75		25	25
Sample control	25(Inactivate)	75		25	25

Table S2. Yield and purity of separated compounds by pH-Zone-refining CCC

		I (Comp1)	II (Comp2)	III (Comp3)	IV (Comp4)
EtOAc (500 mg)	Yield (mg)	6.3	9.5	32.5	19.2
	Purity (%)	95.87	86.38	95.82	92.32
EtOAc (1 g)	Yield (mg)	25.9	29.4	98.2	36.5
	Purity (%)	95.21	91.00	94.48	90.70
BuOH (500 mg)	Yield (mg)	32.5	11.8	4.7	2.1
	Purity (%)	95.52	93.63	88.21	88.78
BuOH (1 g)	Yield (mg)	65.0	24.5	9.3	4.7
	Purity (%)	92.54	95.67	86.63	87.70

Ethyl acetate Fraction

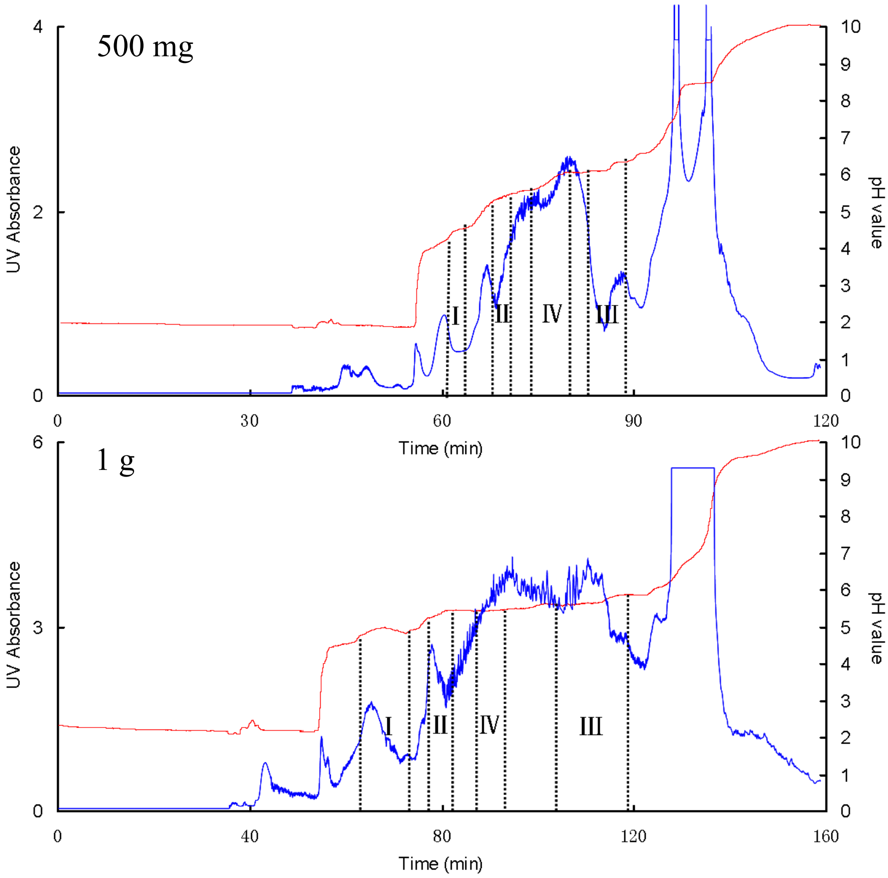

Figure S1. pH-zone-refining CCC of the ethyl acetate fraction of G. divaricate extract. Solvent system: MtBE: n-butanol: acetonitrile: water (1:3:1:5, v/v), TFA (10 mM, pH=2) as retainer in the upper stationary phase and NH_4 OH(10 mM, pH=10.0) as eluter in the lower mobile phase; Flow-rate: 2.0 mL/min; Revolution speed: 1600 rpm; Column temperature: 30 ℃; Detection: 254 nm.

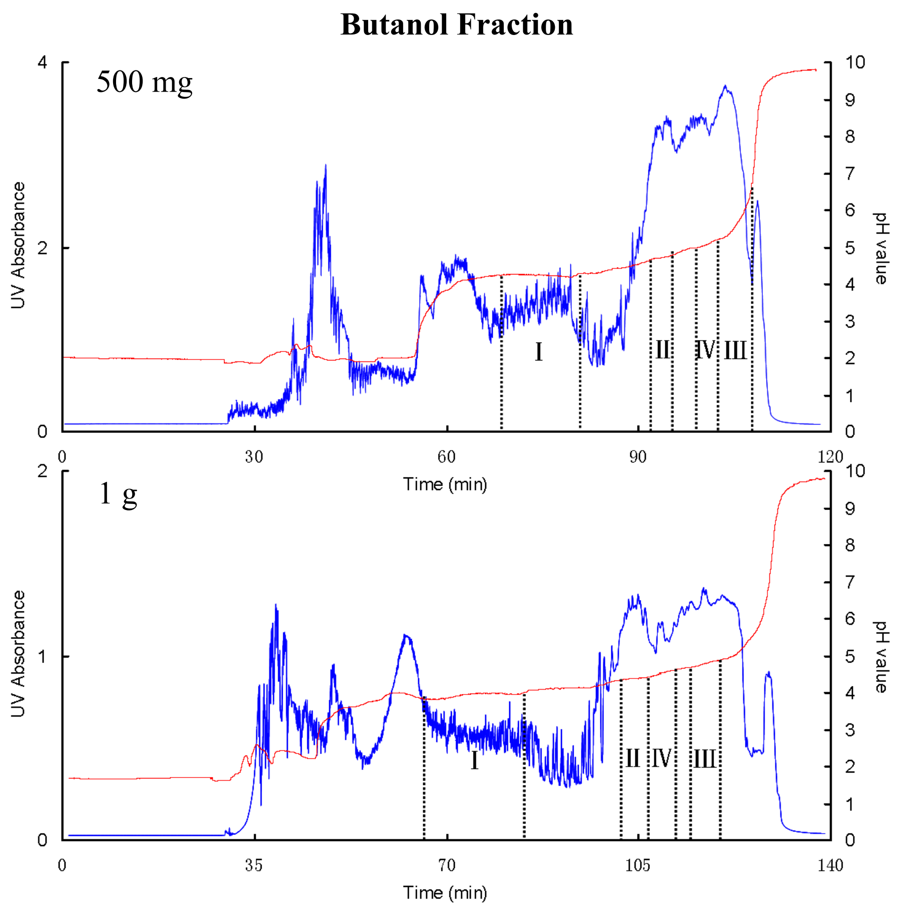

Figure S2. pH-zone-refining CCC of the butanol fraction of G. divaricate extract. The conditions were the same as in Figure 1S.

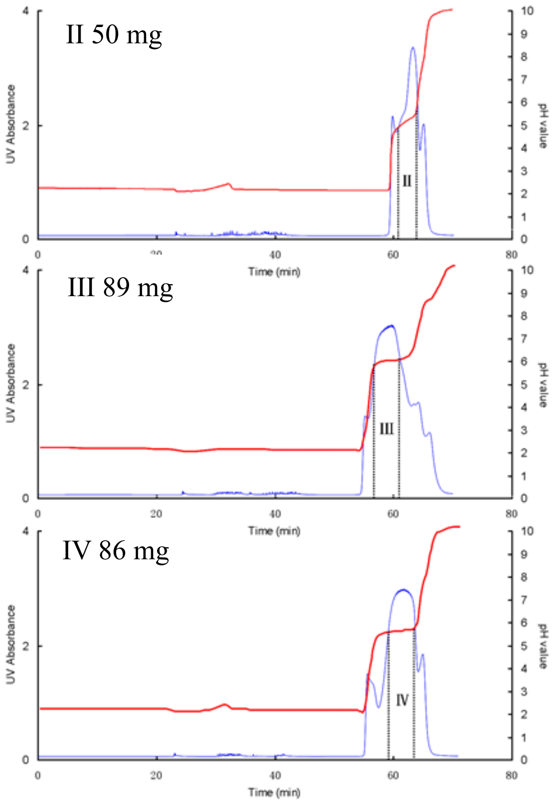

Figure S3. Second purification of three dicaffeoylquinic acids by pH-zone-refining CCC. The conditions are the same with Figures S1 and S2.

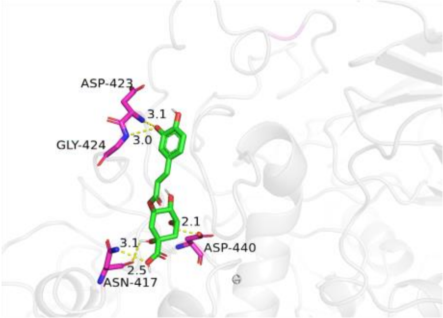

Chlorogenic acid (**1**)

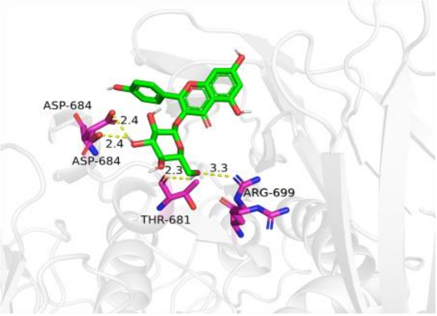

Kaempferol-3-O-β-D-glcopyranoside (**5**)

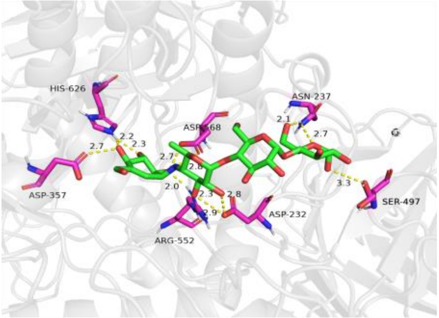

Acarbose

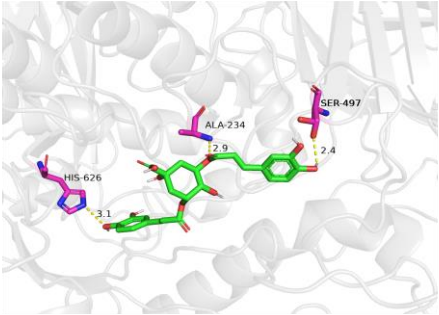

3,4-Dicaffeoylquinic acid (**2**)

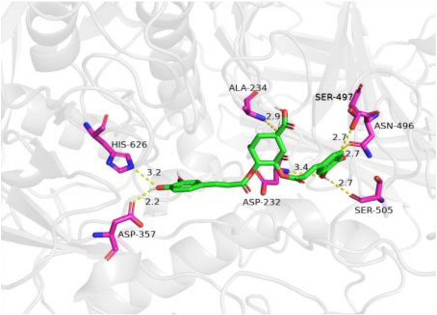

3,5-Dicaffeoylquinic acid (**3**)

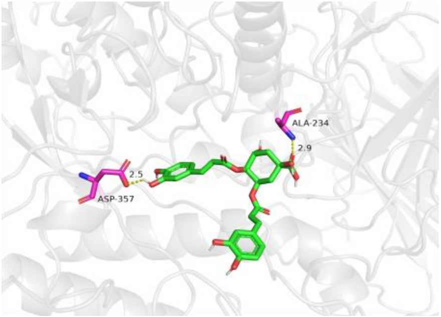

4,5-Dicaffeoylquinic acid (**4**)

Figure S4. Molecular docking sites between the isolated compounds and the α-glucosidase

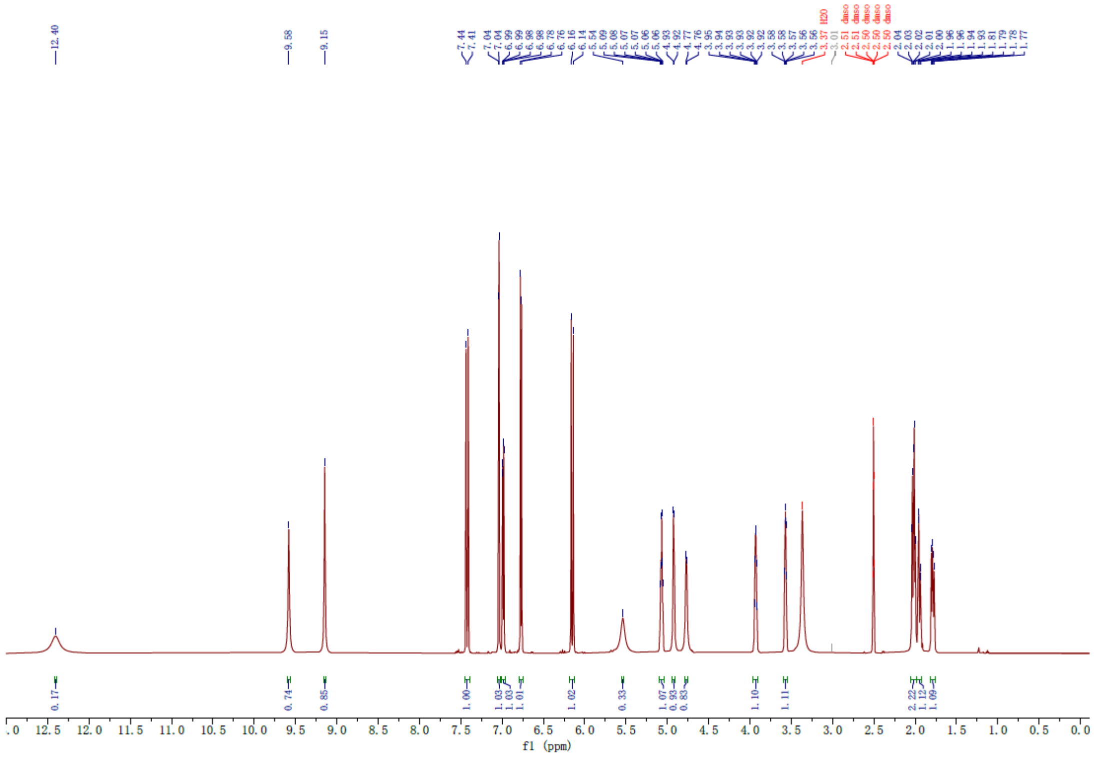

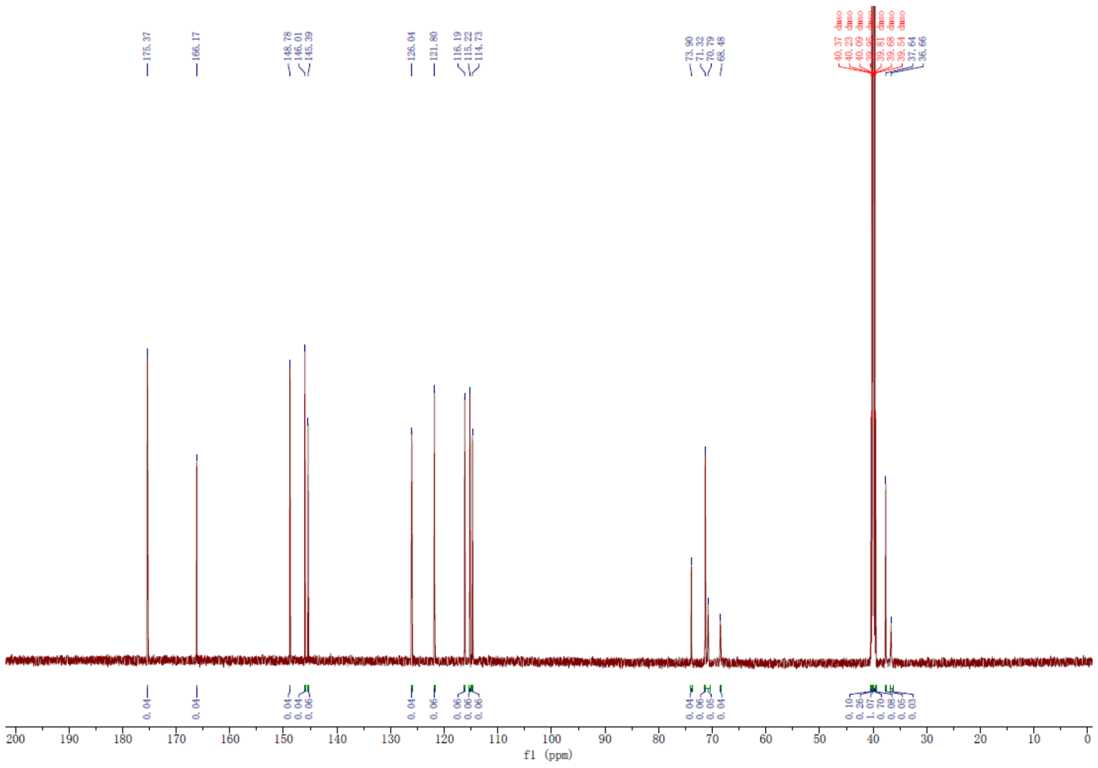

Figure S5-1 ^{1}HNMR and ^{13}CNMR spectrum of Compound **1**

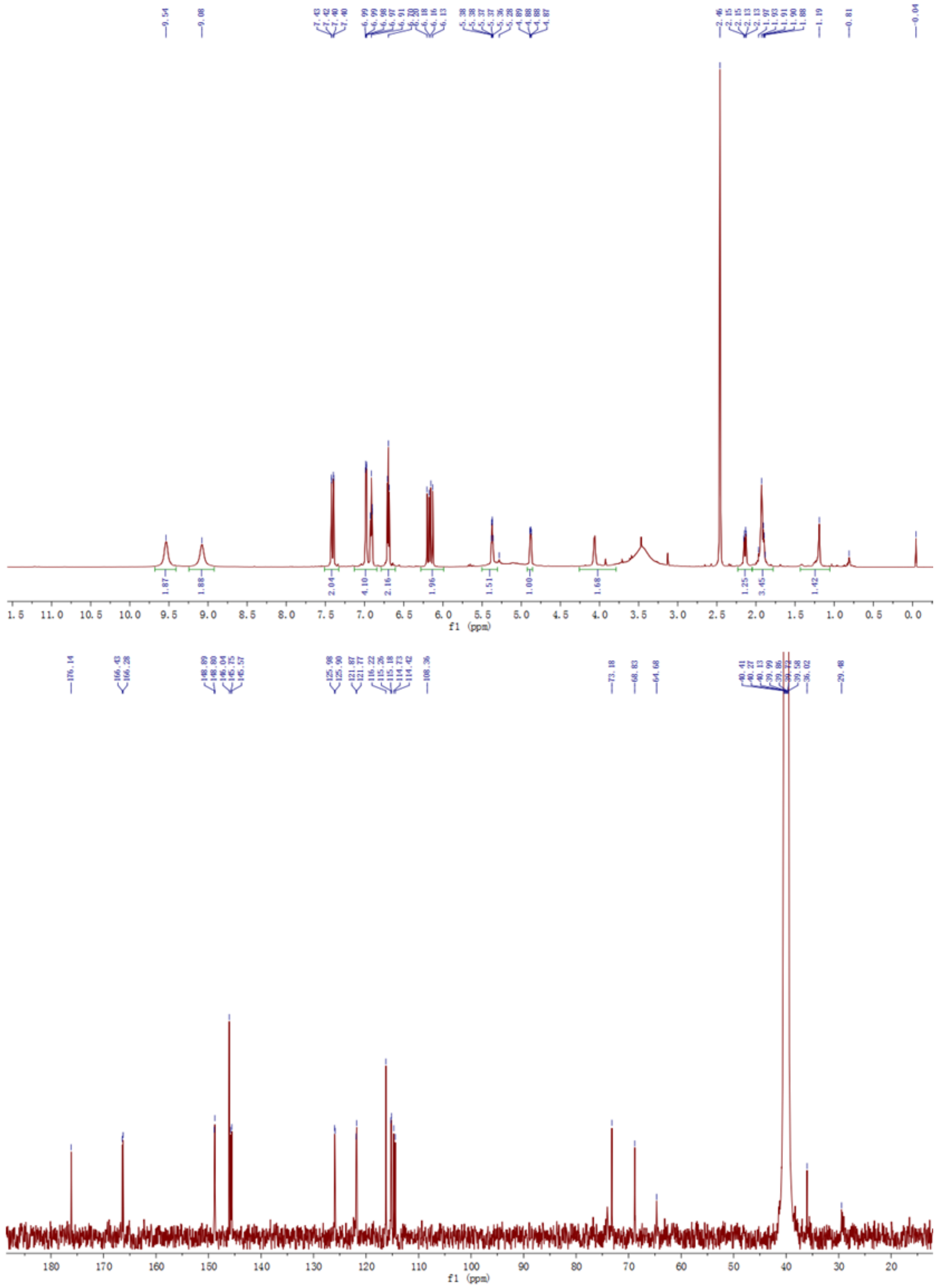

Figure S5-2 ^{1}HNMR and ^{13}CNMR spectrum of Compound **2**

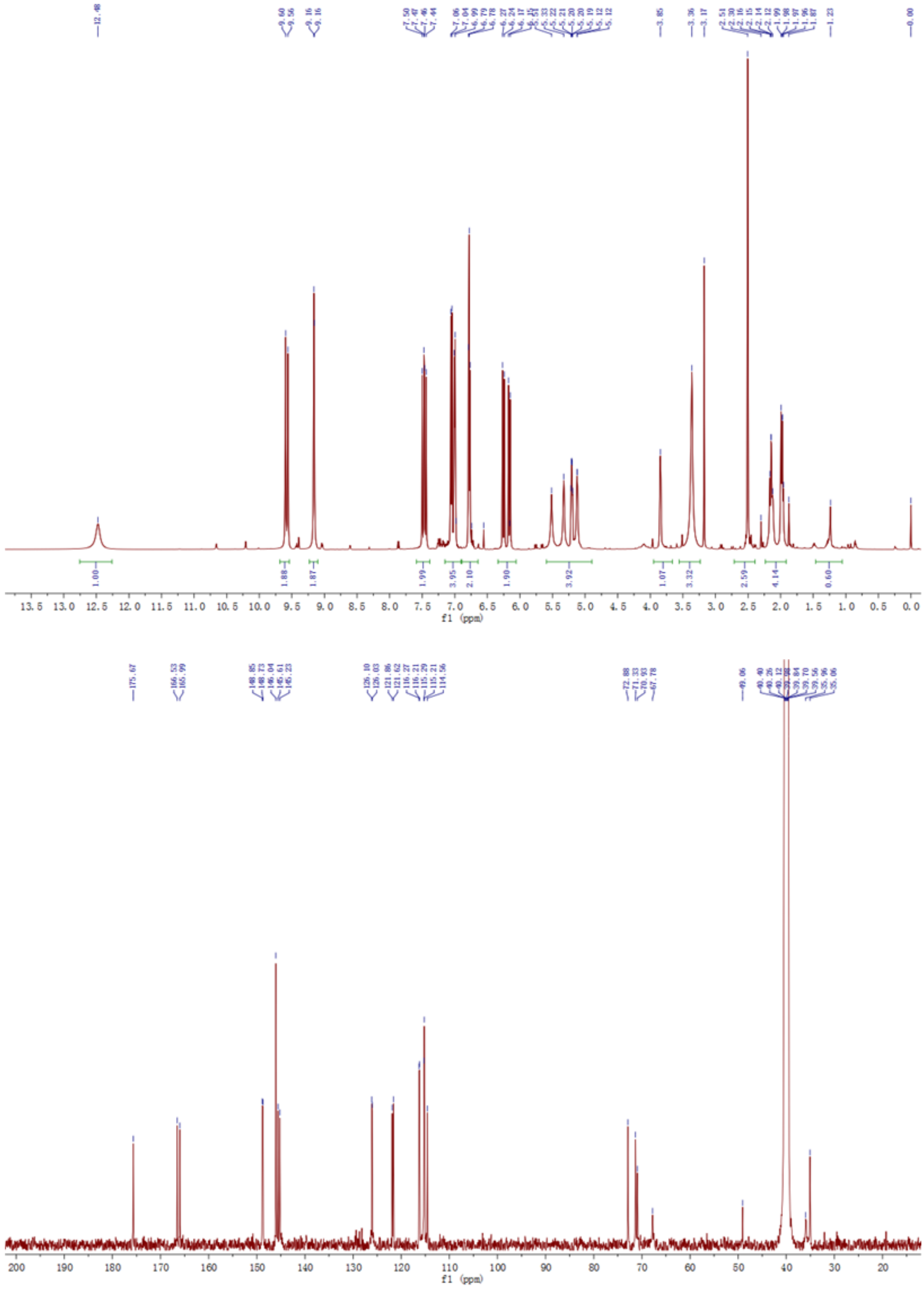

Figure S5-3 ^{1}HNMR and ^{13}CNMR spectrum of Compound **3**

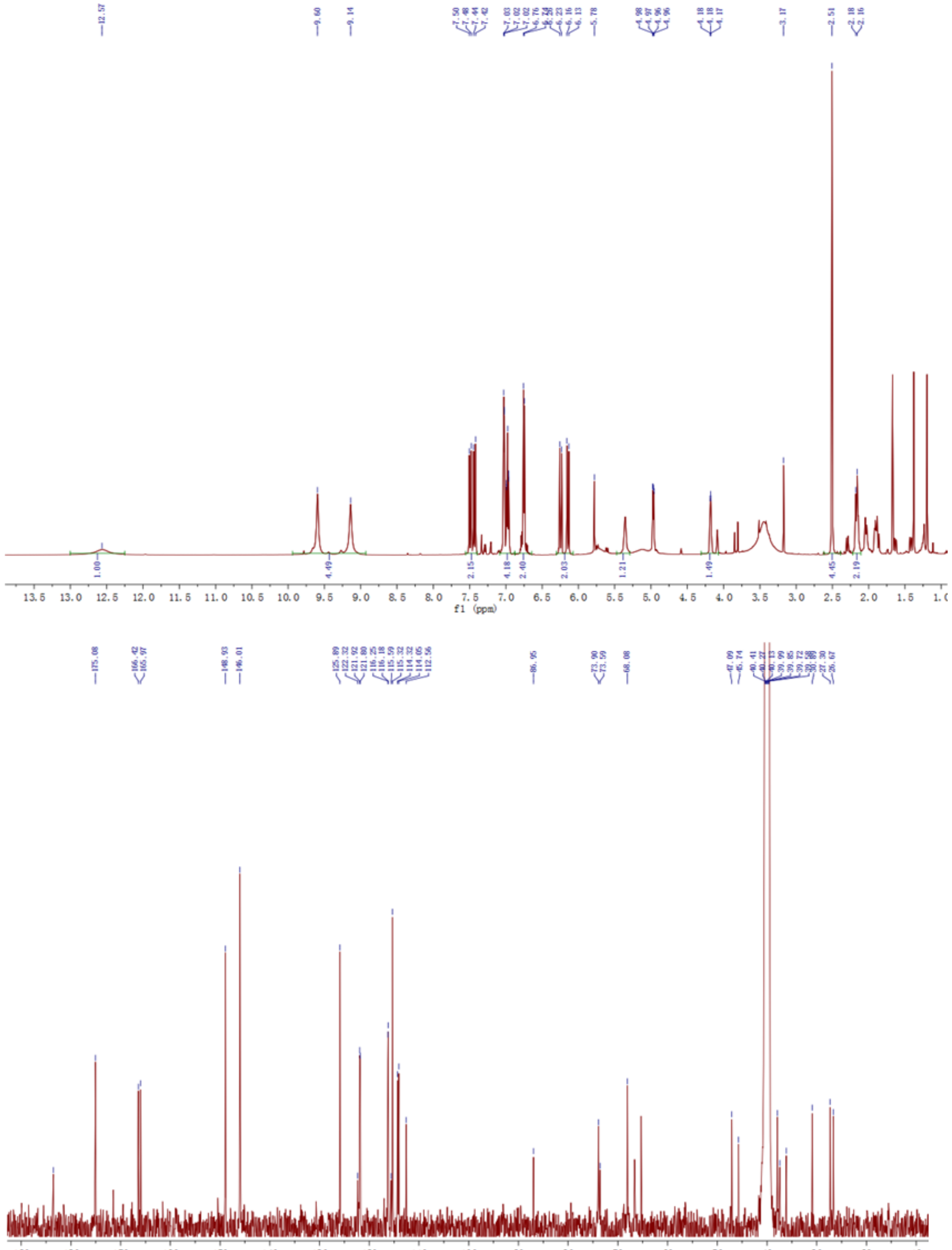

Figure S5-4 ^{1}HNMR and ^{13}CNMR spectrum of Compound **4**

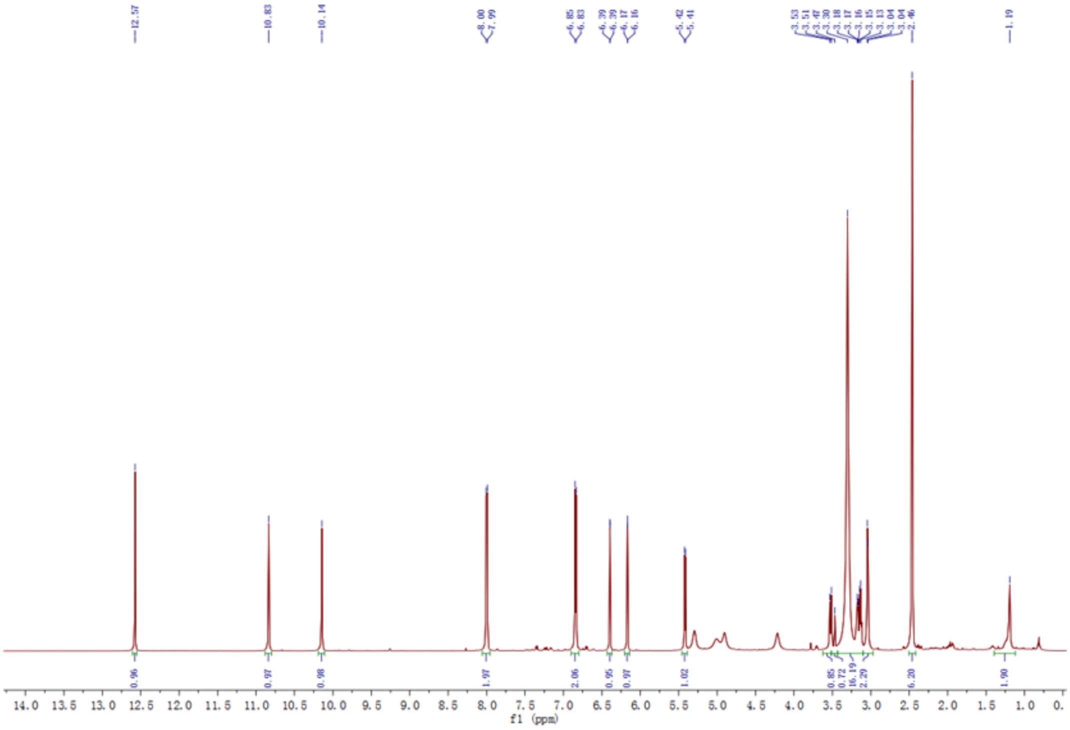

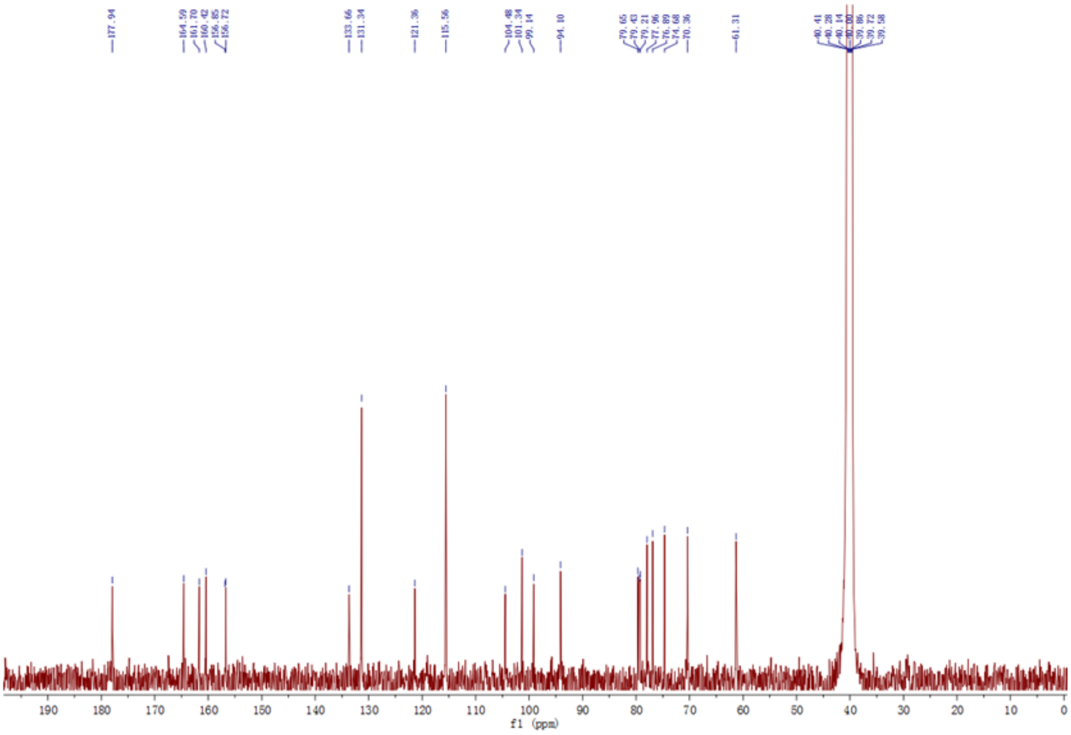

Figure S5-5 ¹HNMR and ¹³CNMR spectrum of Compound **5**
